# Supplementary material for: Competition-Induced Binding Spherical Nucleic AcidFluorescence Amplifier for the Detection of Di (2-ethylhexyl) Phthalate in the Aquatic Environment
Source: Nanomaterials (Basel). 2022 Jun 26;12(13):2196. doi: 10.3390/nano12132196 (PMC9268500; doi:10.3390/nano12132196)
Supplement: Supplementary file 1 [file nanomaterials-12-02196-s001.zip › nanomaterials-1781305-supplementary.pdf]

nanomaterials

Supplementary Materials

Competition-Induced Binding Spherical Nucleic Acid Fluorescence Amplifier for the Detection of Di (2-ethylhexyl) Phthalate in the Aquatic Environment

Lin Yuan †, Dandan Ji *,†, Qiang Fu and Mingyang Hu

College of Environmental Science and Engineering, Qilu University of Technology (Shandong Academy of Science), Jinan 250353, China; m17862975991@163.com (L.Y.); jnhmy17854118089@163.com (Q.F.); micro7306@163.com (M.H.)

* Correspondence: jdd@qlu.edu.cn; Tel: +86-0531-8963-1680

† These authors contributed equally to this work and should be considered co-first authors.

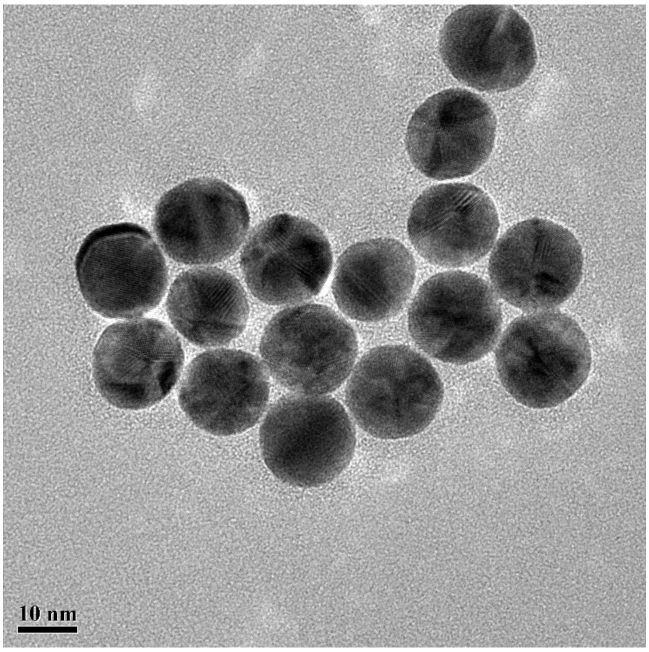

Figure S1. TEM image of 20nm AUNPs.

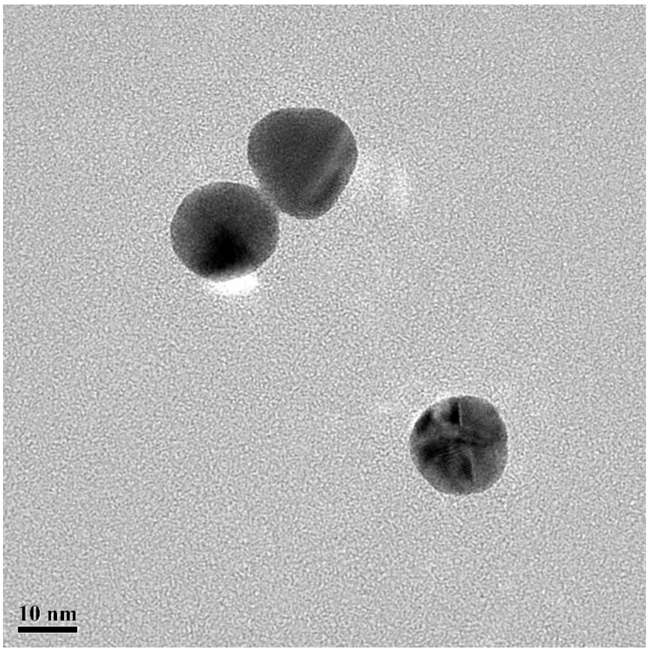

Figure S2. TEM image of SNA.

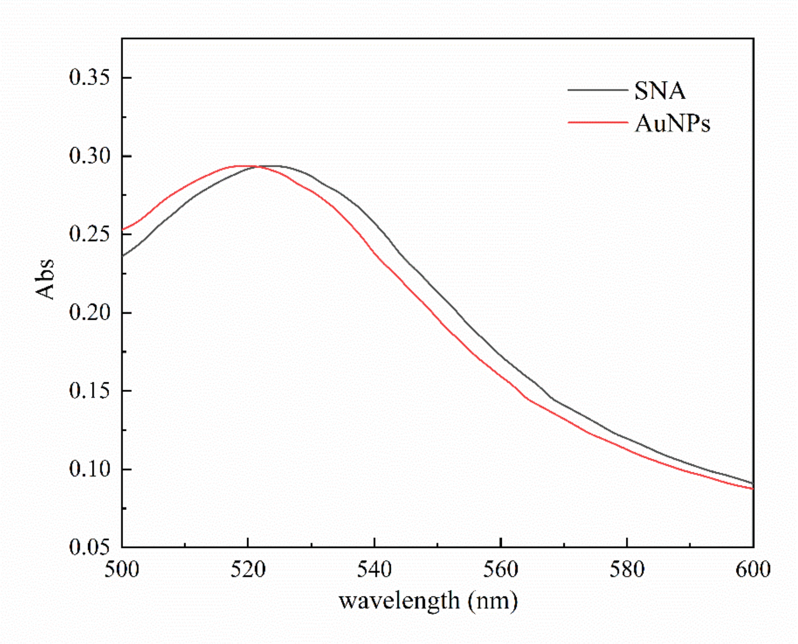

Figure S3. The UV absorption spectra of AuNPs and SNA.
